# Cardiorespiratory and metabolic consequences of detraining in endurance athletes

**DOI:** 10.3389/fphys.2023.1334766

**Published:** 2024-01-22

**Authors:** Arianna Barbieri, Andrea Fuk, Gabriele Gallo, Daniel Gotti, Andrea Meloni, Antonio La Torre, Luca Filipas, Roberto Codella

**Affiliations:** ^1^ Department of Biomedical Sciences for Health, Università degli Studi di Milano, Milano, Italy; ^2^ Department of Movement, Human and Health Sciences, University of Rome Foro Italico, Rome, Italy; ^3^ Department of Neuroscience, Rehabilitation, Ophthalmology, Genetics, Maternal and Child Health, University of Genoa, Genoa, Italy; ^4^ Department of Endocrinology, Nutrition and Metabolic Diseases, IRCCS MultiMedica, Milano, Italy

**Keywords:** deconditioning, training cessation, training reduction, detraining effect, endurance

## Abstract

**Background:** A training program can stimulate physiological, anatomical, and performance adaptations, but these improvements can be partially or entirely reversed due to the cessation of habitual physical activity resulting from illness, injury, or other influencing factors.

**Purpose:** To investigate the effects of detraining on cardiorespiratory, metabolic, hormonal, muscular adaptations, as well as short-term and long-term performance changes in endurance athletes.

**Methods:** Eligible studies were sourced from databases and the library up until July 2023. Included studies considered endurance athletes as subjects and reported on detraining duration.

**Results:** Total cessation of training leads to a decrease in VO_2_max due to reductions in both blood and plasma volume. Cardiac changes include decreases in left ventricular mass, size, and thickness, along with an increase in heart rate and blood pressure, ultimately resulting in reduced cardiac output and impaired performance. Metabolically, there are declines in lactate threshold and muscle glycogen, increased body weight, altered respiratory exchange ratio, and changes in power parameters. In the short term, there is a decrease in insulin sensitivity, while glucagon, growth hormone, and cortisol levels remain unchanged. Skeletal muscle experiences reductions in arterial-venous oxygen difference and glucose transporter-4. Implementing a partial reduction in training may help mitigate drastic losses in physiological and performance parameters, a consideration when transitioning between training seasons.

**Conclusion:** There is a dearth of data investigating the detraining effects of training reduction/cessation among endurance athletes. Delving deeper into this topic may be useful for professionals and researchers to identify the optimal strategies to minimize these effects.

## 1 Introduction

Regular and consistent endurance exercise training stimulates physiological, anatomical and performance adaptations, which may undergo partial or total reversal due to cessation or reduction of habitual physical activity caused by illness, injury or other factors (Mujika and Padilla, 2000b). The principle of reversibility of training is equivalent to the principle of detraining (Mujika and Padilla, 2001b). Detraining has been defined as the partial or total loss of training-induced adaptations, consequent to the reduction or complete cessation of physical activity (Mujika and Padilla, 2000b). In agreement with Mujika and Padilla’s, this review divides detraining into:

- Short-term; when the period of stopping or reducing training is less than or equal to 4 weeks;
- Long-term; when detraining extends for a period longer than 4 weeks.

The topic of detraining in athletes has been addressed by systematic reviews and meta-analyses devoted to examining data regarding modifications and adaptations to deconditioning. In particular, the most recent ones have dealt with VO_2_max (Zheng et al., 2022) and cardiac effects (Petek et al., 2022). Since 2000, Mujika and Padilla’s studies were dealing with the analysis of detraining on multiple levels (cardiorespiratory, metabolic, muscular and hormonal) in the short and long term, comparing highly trained athletes from any sport discipline with recently trained individuals (Mujika and Padilla, 2000a; Mujika and Padilla, 2000b).

The emergence of the COVID-19 pandemic prompted the interest in crafting this review to delve deeper into this subject. This investigation involved a thorough review and analysis of scientific literature, spanning from earlier works to recent publications. This study was also focused on specific subjects: endurance athletes. In fact, due to the COVID-19 pandemic, National and International authorities declared a state of emergency and took restrictive measures, such as closing non-essential activities and requiring people to stay home, to reduce the risk of infection (Girardi et al., 2020; Paoli and Musumeci, 2020). These restrictions exposed most athletes to the risk of detraining, with a sudden and unexpected cessation of both competitions and training, and a substantial cutback in their daily physical activities (Girardi et al., 2020; Paoli and Musumeci, 2020). In many countries, such as Italy and Spain, this period lasted for at least 8 weeks (Girardi et al., 2020; Paoli and Musumeci, 2020).

The objective of this systematic review is to comprehensively outline the repercussions linked with both short- and long-term detraining in endurance athletes. Furthermore, this review aims to offer potential avenues for future scientific research.

## 2 Methods

This systematic review was conducted according to PRISMA (Preferred Reporting Items for Systematic reviews and Meta-Analyses) guidelines. Bibliographic research was conducted using databases such as PubMed, Scopus, Web of Science and Medline and by consulting library articles.

### 2.1 Search strategy

The Bolean search method was employed to refine search results to be focusing on documents containing key terms central to this review’s scope. The main keywords used in records and databases include “detraining,” “training cessation,”, “training reduction”, “deconditioning,” “athletes,” “endurance,” “performance,” “cardiovascular remodeling,” “body composition,” “VO_2_max,” “lactate,” “metabolic/muscular characteristics,” “hormonal adaptation,” and “physical performance."

### 2.2 Selection criteria

Studies were deemed eligible for inclusion if they met the following criteria: (a) detailed reporting of sampling and screening procedures including data collection, study design, and results; (b) documentation of the detraining duration in the paper; (c) comparison of results between pre- and post-detraining for the main values analyzed by the study; and (d) the subjects were classified as endurance-trained athletes (performance level 3 (Pauw et al., 2013; Decroix et al., 2016) reporting minimum VO_2_max levels for female, (absolute ≥3 L min^–1^ or relative ≥48 mL min^–1^ · kg^–1^) mixed and male groups (absolute ≥4.2 L min^–1^ or relative ≥55 mL min^–1^ kg^–1^), if tested, otherwise ≥5 endurance training hours or sessions per week with at least 2 years of experience (e.g., VO_2_max not measured, master athletes >50 years old). In cases where the duration of training years was not explicitly reported, athletes were categorized based on their status as elite or Olympic level. Subjects encompassed a diverse range and were not limited by age, sex, or level of competition. All participants were engaged in endurance sports such as running, cycling, swimming, kayaking, skiing, ski mountaineering, mountain running, rowing or triathlon.

There were no limitations on the publication date for inclusion. However, studies presenting information in an unclear or ambiguous manner were excluded from the review.

## 3 Results

### 3.1 Study selection

**FIGURE 1:**
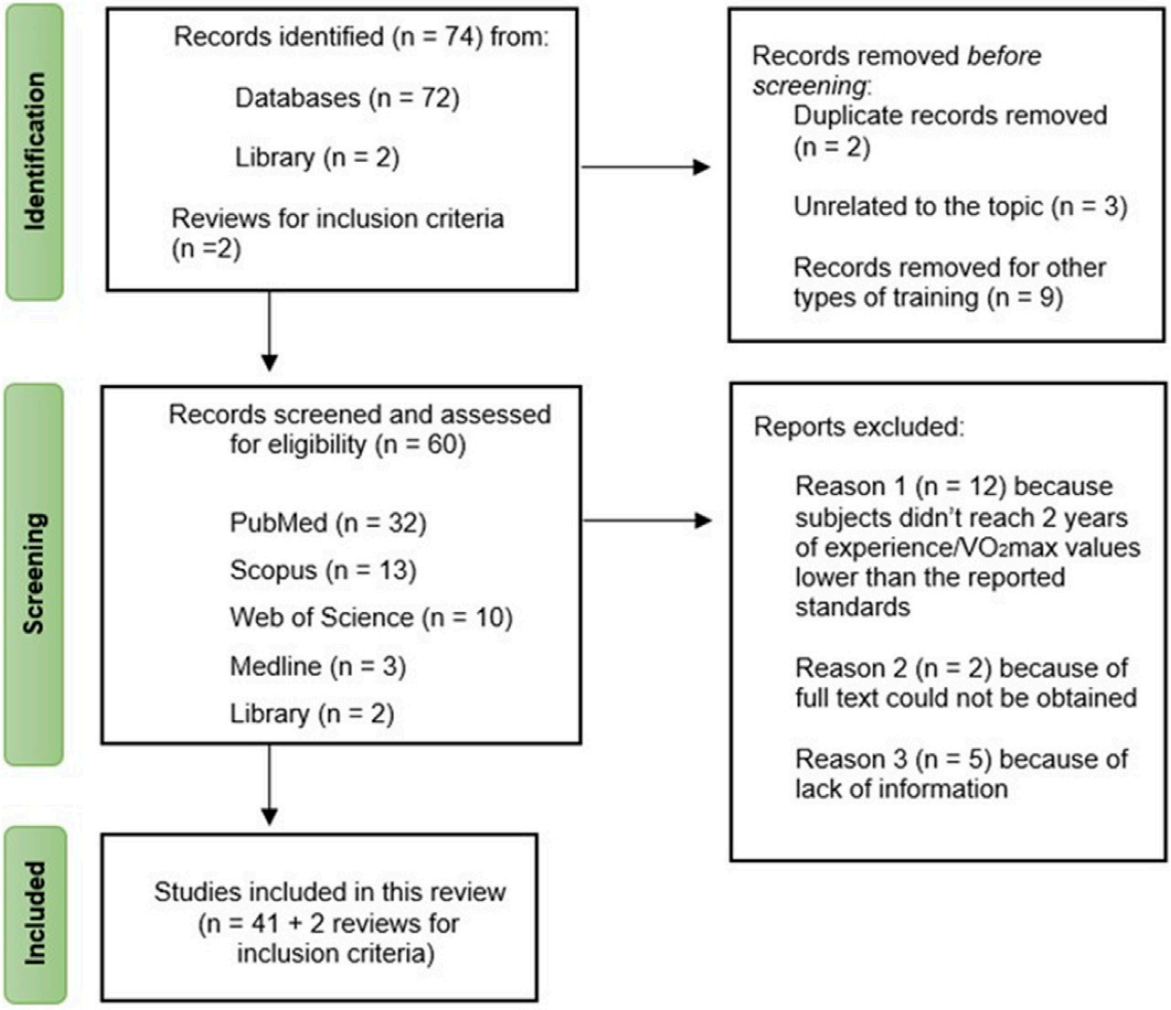
Flow chart of the study selection process.

Through database and library searches, two reviews were identified as useful resources for establishing inclusion criteria. Initially, 74 studies were discovered, but after screening based on title and abstract, 14 studies were excluded: 9 studies focused on other types of training (e.g., resistance training, strength training, multicomponent training), 3 studies were unrelated to the review’s focal topic, and 2 studies were duplicates of previously selected articles. Consequently, 60 studies remained. However, only 41 of these met the inclusion criteria, while 19 studies were excluded for various reasons (e.g., subjects not meeting the required 2 years of experience and/or having VO_2_max values lower than reported standards, lack of sufficient information). The study selection process is outlined in Figure 1.

### 3.2 Extraction of data

**FIGURE 2:**
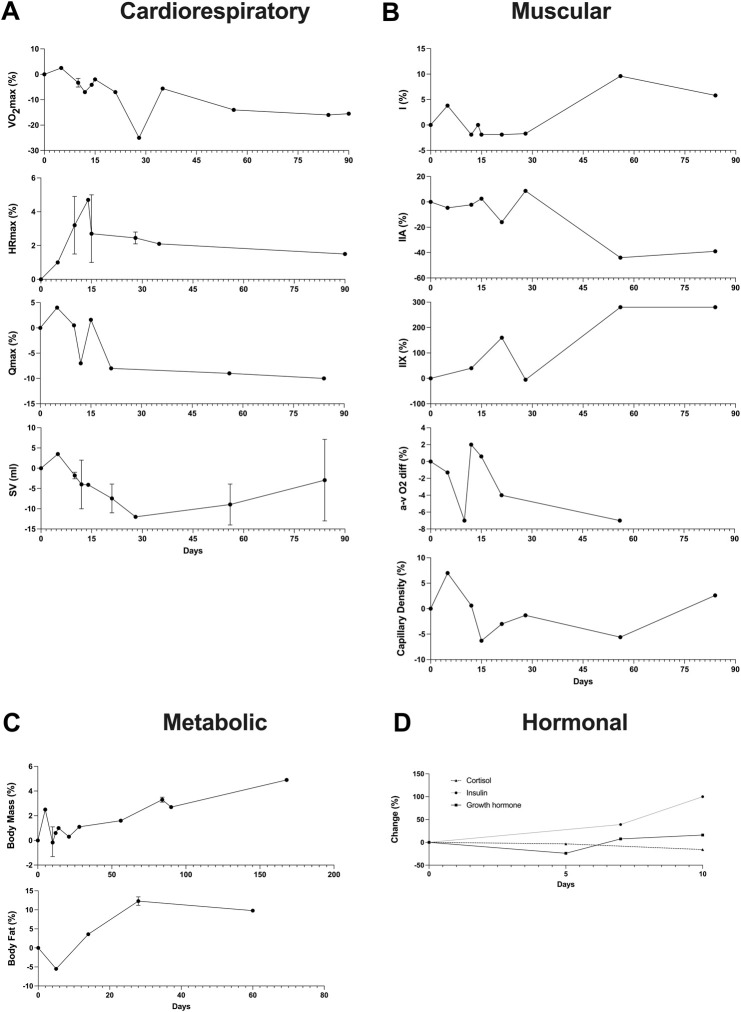
Timeline of changes in the main variables after a detraining period. In instances where multiple data points exist within the same time interval, the average value was reported alongside the maximum and minimum. **(A)** Cardiorespiratory variables (VO_2_max, HRmax, Qmax, SV); **(B)** Muscular variables (Fiber types, a-v O_2_ diff, Capillary Density); **(C)** Metabolic variables (Body Mass, Body fat); **(D)** Hormonal variables (Cortisol, Insulin, Growth hormone).

For the 41 studies included in the review, a data extraction table (Supplementary Table S1) was compiled. This table delineated the duration of detraining expressed in days, classified the type of detraining as total cessation or partial reduction, outlined the main parameters considered, detailed the pre- and post-detraining parameter values, and calculated the percentage difference between these values. Additionally, to illustrate the effects of short-term *versus* long-term training cessation on primary variables, a summary table (Table 1) was created. Furthermore, a visual representation of the timeline depicting changes in the main variables following a detraining period was presented in Figure 2.

**TABLE 1:** Short-term vs. Long-term effects of training cessation on main variables.

Variables	Short-term detraining (0–4 weeks)	Long-term detraining (>4 weeks)
	*Cardiorespiratory*	
VO_2_max (L*min^−1^)	−25.5% < x < +2.4%	−16% < x < −11.3%
HRmax (beats*min^−1)^	+1% < x < +5%	+1% < x < +1.5%
Qmax (L*min^−1^)	−8% < x < +4.4%	−10% < x < −9%
SV (ml)	−12% < x < +3.4%	−14% < x < −13%
LVM (g)	−19.5% < x < −1.8%	−23.8% < x < 19.5%
LV PWT (cm)	−12.5%	−25% < x < −12%
DBP (mmHg)	−4%	−4.6% < x < +2.8%
SBP (mmHg)	0%	−0.7% < x < +3.6%
LA-peak (mmol*L^−1^)	−23%	+11.4%
	*Metabolic*	
Body mass (kg)	+0.3% < x < +8.7%	+1.6% < x < +11.3%
Body fat (%)	−5.5 < x < 11.2	+9.8% < x < +28.8%
	*Muscular*	
a-v̄̄̄ O_2_ diff (ml*100 ml^−1^)	−1.3% < x < +2%	−7% < x < −4%
Capillary density	−6.3% < x < +7.1%	−5.6% < x < +2.6%
Fiber		
I (%)	−1.9% < x < +3.8%	+5.8% < x < +9.6%
IIA (%)	−16.3% < x < +8.7%	−39% < x < −44%
IIX (%)	−5.3% < x < +160%	+160% < x < +280%
	*Hormonal*	
Insulin	+7% < x < +39%	NA
Cortisol	−24% < x < +83%	NA
Growth hormone	−18% < x < −3.3%	NA

### 3.3 Cardiorespiratory detraining

#### 3.3.1 Maximal oxygen uptake

VO2max refers to the maximum amount of oxygen that muscles can take in, absorb, and use during intense endurance exercise within a specific time frame. It is widely regarded as the gold standard for measuring cardiorespiratory fitness (Zheng et al., 2022).

Training is an effective means of preserving and enhancing VO_2_max, albeit with variable outcomes influenced by both intensity and volume. Genetics largely determine VO_2_max, with improvements typically ranging from 15% to 30%. Remarkably, elite athletes often achieve values exceeding 90 mL/kg/min. Research suggests that high volume training may foster future potential enhancements, while high-intensity training appears to elicit more rapid adaptations (Buchheit and Laursen, 2013). However, Attention must be given to the reversibility of VO_2_max adaptability, signifying its return to pre-intervention values upon cessation or significant reduction of training stimuli (Zheng et al., 2022). Upon the cessation or reduction of training, a significant decline in VO_2_max becomes apparent within just a few days of commencing detraining.

Coyle et al., reported a −7% decline of VO_2_max in a group of runners and cyclists just 12 days later training interruption (Coyle et al., 1984). In a group of distance runners, 14 days of training cessation, caused −4.7% losses when compared with the training state (Houmard et al., 1992); similar results were observed after 15 days of total stop (−3.7%) (Chen et al., 2022). In contrast with previous studies, Cullinane et al. found no change in VO_2_max after 10 days of training cessation (Cullinane et al., 1986): this result may be explained by the variability of daily physical activity, not specifically related to sports, performed by athletes during the period of detraining (Mujika and Padilla, 2001b). When inactivity is prolonged up to 3 weeks, there is a decrease of 4.18% (Martin et al., 1986). After 5 weeks there is a decrease of 10.1% (García-Pallarés et al., 2009), following 56 days a decrease of 13% (Coyle et al., 1984; Coyle et al., 1985) and after 2 months a decrement of 20% (Martin et al., 1986). No further significant decrements are found after 12 weeks (Coyle et al., 1985; Martin et al., 1986).

The decline in VO_2_max observed between the 21st and 84th day of detraining seems attributed to a reduction in the maximum arterial-venous oxygen difference (Coyle et al., 1984). The drop in VO_2_max following brief periods of detraining (less than 4 weeks) is primarily due to the decrease in total blood and plasma volume (Mujika and Padilla, 2001b). This reduction significantly impacts performance (Doherty et al., 2003).

When considering detraining as a partial reduction in training load (by both volume and intensity), VO_2_max levels seems to be maintained after 4-week of low-volume and moderate-to-high intensity training (Madsen et al., 1993; McConell et al., 1993). In contrast, Garcia-Pallares reported a loss of–5.6% in VO_2_max after 5 weeks of reduced training volume at moderate intensity in a group of elite kayakers (Martin et al., 1986).

Recent studies exploring the consequences of partial training reduction indicate that maintaining VO_2_max levels is achievable by incorporating a single weekly high-intensity training (HIT) session alongside reduced low-intensity training (LIT) volume during an 8- week transition period. Conversely, using solely LIT during a partial cessation period, would not produce the same conservative effect (Rønnestad et al., 2014). Incorporating a weekly sprint session within a constrained LIT volume yields no significant alterations in VO_2_max when compared to exclusively performing LIT over a shorter span of 3 transition training weeks (Almquist et al., 2020).

#### 3.3.2 Blood volume

The decline of VO_2_max is related to the reduction in blood volume and in plasma volume. According to Coyle et al., a period of complete detraining lasting between 2 and 4 weeks during orthostatic exercise (cycling on a stationary ergometer) resulted in a 9% reduction of blood volume, attributed to a 12% decline in plasma volume (Coyle et al., 1986). Houmard et al. presented a 5.1 %± 1.9% decrease in resting plasma volume following 14 days of inactivity (Houmard et al., 1992).

The decrease in blood volume significantly impacts cardiovascular regulation during exercise, likely influencing the low-pressure baroreceptors responsible for managing venous blood return to the heart. Consequently, there is a reduction in central venous pressure, triggering vasoconstriction in the splanchnic area, skin, and inactive muscles. This phenomenon potentially elucidates the observed increases in mean arterial pressure (Coyle et al., 1985).

#### 3.3.3 Heart rate

Because of blood volume’s decline, there is an increase in maximal and submaximal heart rate following periods of training reduction or total cessation.

In short-term detraining, maximal heart rate (HRmax) increases by 2-9 beats per minute (Coyle et al., 1984; Cullinane et al., 1986; Houmard et al., 1992; Doherty et al., 2003; Chen et al., 2022) while submaximal heart rate is distinguished between orthostatic and supine exercise: in the first case, there is an increase of 2.6% after 21 days, of 10%–11% from day 56–84. In supine exercises there is an increase of 4.7%, 5.6%, 11.2% after 12, 56, 84 days respectively (Martin et al., 1986).

After 1 year of total detraining, resting heart rate increased by 13%, an average of 7 beats per minute higher than pre-detraining values (Pelliccia et al., 2002).

The rise in both maximal and submaximal heart rate during short-term detraining can be attributed to a decrease in stroke volume resulting from reduced ventricular filling, which is primarily caused by the decrease in plasma volume (Coyle et al., 1986). Additionally, the increase in peak heart rate can be viewed as a compensatory mechanism to maintain cardiac output despite the decrease in plasma volume (Cullinane et al., 1986).

However, there are some studies that found no change in this parameter after inactivity or reduced training periods of 10 days, 3, 4, and 6 weeks (Cullinane et al., 1986; Petretta et al., 1991; Maron et al., 1993).

#### 3.3.4 Stroke volume and cardiac output

Deconditioning after several years of intense training leads to a reduction, during orthostatic exercise, in stroke volume, calculated as the ratio of cardiac output to heart rate (Martin et al., 1986). On the other hand, during supine exercise no significant differences are found (Martin et al., 1986). The variance between orthostatic and supine positions could be attributed to pressure alterations resulting from position changes, consequently impacting venous return. Differently, a few days after training cessation, a reduction of stroke volume during orthostatic exercise can be registered: after 10 days −0.9% (Doherty et al., 2003), −4.1% after 2 weeks (Chen et al., 2022)and −14% after 56 days of inactivity (Coyle et al., 1984).

The reduction in stroke volume during upright exercise is linked to a decrease in the telediastolic dimension of the Left Ventricle (Martin et al., 1986) and does not appear to be due to a deterioration of myocardial contractility (Coyle et al., 1986; Martin et al., 1986).

As for cardiac output, it appears to remain unchanged after 5–10–15 days of detraining (Doherty et al., 2003): The maintenance of cardiac output in the absence of change appears to be influenced by the typical relationship between HR and SV, as observed during short-term detraining where an increase in HR coincides with a decrease in stroke volume. After 21 days, a decline of 8% is recorded, not falling beyond this decline as detraining days progressed (Coyle et al., 1984).

#### 3.3.5 Blood pressure

Following short-term training cessation, mean blood pressure increases by 7% during submaximal upright exercise (Coyle et al., 1986). Conversely, after long-term detraining, there is an 11% increase in blood pressure during orthostatic exercise and a 1% increase in supine positions (Martin et al., 1986). This rise indicates a concurrent increase in total peripheral resistance (TPR) by 7%–8% (Coyle et al., 1986; Petretta et al., 1991). Moreover, even after 12 weeks of detraining, TPR remains within the range of a 7.9%–9% increase (Martin et al., 1986).

#### 3.3.6 Cerebral blood flow

Cerebrovascular system responds to insufficient training stimulus: in master athletes, after 10 days of inactivity, there is a decline in resting cerebral blood flow (rCBF) at 8 regions of the brain: inferior temporal gyrus, fusiform gyrus, inferior parietal lobule, cerebellar tonsil, lingual gyrus, precuneus, bilateral cerebellum and right and left hippocampus (Alfini et al., 2016). After short-term detraining, a decrease in cortical and hippocampal cerebral blood flow is observed, despite participants showing no changes in cognitive function in the study. However, these effects might significantly influence brain function in older individuals by altering arterial transit time or blood volume in the brain (Alfini et al., 2016).

#### 3.3.7 Cardiac dimensions

A regular aerobic training regimen induces cardiac adaptations, including the augmentation of left ventricular size, mass, and thickness, which play a crucial role in health, longevity, and sports performance. However, cessation or reduction of daily physical activity leads to the loss of these trained heart adaptations (Petek et al., 2022).

After short-term inactivity (12 weeks of detraining), total peripheral resistance (TPR) increase by 7%–8% remaining in the range of 7.9%–9% increase (Coyle et al., 1986; Martin et al., 1986; Petretta et al., 1991).

##### 3.3.7.1 Left ventricular (LV) remodeling

Several weeks following the initiation of detraining, notable reductions in LV mass are observed. While most studies have documented regressions of LV mass (Cullinane et al., 1986; Martin et al., 1986; Petretta et al., 1991; Maron et al., 1993), changes in LV size do not consistently decrease during deconditioning (Petek et al., 2022).

The telediastolic dimension of LV is calculated at the beginning of the QRS complex of the electrocardiogram, and it is identified as the distance between the posterior endocardial wall and the interventricular septum in its left portion (Martin et al., 1986).

At 10 days of training cessation, LV telediastolic dimension is unchanged (Martin et al., 1986), whereas a change is instead observed from the third week. Significant reduction also occurs involving LV cavity: 9 athletes of the 40 considered maintain the persistent dilatation during the detraining period, while the others show a reduction in LV telediastolic cavity (Pelliccia et al., 2002).

On the other hand, the telesystolic ventricular dimension, is the shortest perpendicular distance from the posterior wall of the endocardium to the left side of the interventricular septum (Martin et al., 1986). Martin et al. report analyses of LV dimensions during supine and orthostatic exercise: in supine exercises there is an increase of 3.4% after 56 days of detraining, while in orthostatism there is a decrease of 6% and 10% after 21 and 56 days of inactivity respectively (Martin et al., 1986). Pelliccia et al. record a 6.5% decline following 1 year of complete detraining in 40 endurance athletes (Pelliccia et al., 2002).

There are studies that reported, because of detraining, reductions in LV maximum wall thickness, intraventricular septum and LV wall thickness (Cullinane et al., 1986; Petretta et al., 1991; Maron et al., 1993; Giada et al., 1998). Regarding the latter measure, there are mixed results concerning the extent and time of thinning (Petek et al., 2022). Martin et al. (1986) find no major changes in wall thickness after 3 weeks of detraining: significant reductions are shown following 8 weeks of detraining. However, in 18 rowers declines in wall thickness are documented after 1 week of drastic training reduction, with no further declines in the subsequent 3 weeks (Petretta et al., 1991). According to other studies, maximum wall thickness can decrease by 9% after 6 weeks (Maron et al., 1993) and by 15% with training cessation longer than 1 year (Pelliccia et al., 2002).

Regarding the influence of age on cardiovascular adaptation to endurance training and to 2 months detraining, one study shows that LV wall thickness decreases only in younger athletes (19–25 years old), while LV mass, diameter and telediastolic volume decrease only in athletes aged 50–65 years (Giada et al., 1998).

##### 3.3.7.2 Right ventricular remodeling

Studies concerning right ventricular remodeling are rather limited (Petek et al., 2022). Following long-term detraining, the telediastolic cavity size of the right ventricle appears to decrease by 7% (Pelliccia et al., 2002).

##### 3.3.7.3 Atrial remodeling

Few studies have focused their research on atrial remodeling: long-term (1–13 years) changes in left atrial size and volume have not been identified (Giada et al., 1998; Pelliccia et al., 2002).

#### 3.3.8 Ventilatory function

Regarding changes in ventilatory function, a rapid decline due to the decrease in maximal expiratory ventilatory volume is evident, consequent to short-term training cessation (Houmard et al., 1992; Houmard et al., 1993). During submaximal intensity exercise, an increase in ventilatory volume and ventilatory equivalents is evidenced (DB and HS, 1972; Coyle et al., 1985). These increases indirectly suggest a rise in muscle and blood lactate levels as a consequence of detraining (Houmard et al., 1992). Similar results on maximal ventilatory volume are reported before and after 4 weeks partial training reduction (McConell et al., 1993).

#### 3.3.9 Endurance performance

The reversal of adaptations induced by the cessation or reduction of habitual physical activity leads to a decline in sports performance (Mujika and Padilla, 2000b; Houmard et al., 1992; Houmard et al., 1993), with no observed changes in running economy. Endurance is notably compromised after just 2 weeks of training cessation, evidenced by a 9% decrease in Time To Exhaustion (TTE), with no observed changes in running economy (Houmard et al., 1992). A group of runners showed that they were able to maintain muscle endurance despite 2 weeks of inactivity (Chen et al., 2022). Conversely, over a more extended period of partial training reduction, a cohort of triathlon athletes, proficient in running and cycling, experienced a decline in endurance capacity (specifically, Time To Exhaustion at 75% VO_2_max), reducing it by 21% when engaging in only one weekly low-volume High-Intensity Interval Training (HIT) session (35 min at 95% HRmax) (Madsen et al., 1993).

Short-term detraining has shown an elevation in blood lactate concentration following submaximal exercise (García-Pallarés et al., 2009; Chen et al., 2022). This increase, coupled with altered Mg^2+^ transport from the extracellular to the intracellular compartment, potentially inhibits Ca^2+^ release from the sarcoplasmic reticulum (Głyk et al., 2022). Consequently, this inhibition could impair contractile capacity (García-Pallarés et al., 2009) and be a contributing factor to the reduction in TTE (García-Pallarés et al., 2009) (20).

### 3.4 Metabolic detraining

#### 3.4.1 RER

Respiratory exchange ratio (RER) increases in short-term detraining during submaximal (Coyle et al., 1984; Madsen et al., 1993) and maximal exercises (Houmard et al., 1992). In seven endurance athletes, RER increases from 0.93 of the trained state to 1, after 84 days of detraining, during a submaximal exercise performed at the same absolute intensity (Coyle et al., 1985). These observed changes stem from an increased reliance on carbohydrates, compromising lipid metabolism (Mujika and Padilla, 2000b).

#### 3.4.2 Energy parameters

The primary alteration observed during detraining affects energy parameters in carbohydrate and lipid metabolism. Resting triglyceride concentration decreases with training, attributed to increased fatty acid turnover. Alongside this reduction, there’s an elevation in glycerol and apolipoprotein C3 (Apo-C3) levels. Higher concentrations of Apo-C3 are linked to hypertriglyceridemia and the release of VLDL and chylomicrons into the bloodstream (Petibois and Déléris, 2003).

Following short-term training cessation, there is a decrease in post-exercise glucose concentration (Petibois and Déléris, 2003) and an increase in resting glucose concentration. This rise may be attributed to a decreased rate of glucose disposal resulting from reduced insulin action (McCoy et al., 1994; Arciero et al., 1998; Petibois and Déléris, 2003).

While skeletal muscle exhibits heightened glycolytic utilization during detraining, the hepatic level experiences a decline in both glycogenolysis and glycogenosynthesis (Mikines et al., 1989; Petibois and Déléris, 2003). Inactivity leads to decreased triglyceride levels during exercise and in adipose tissue release, along with reductions in glycerol levels during both activity and rest (Coyle et al., 1985; Mikines et al., 1989; Petibois and Déléris, 2003). Apo-C3 levels tend to revert to pre-training cessation levels. Notably, no changes are observed in hemolytic parameters like haptoglobin and transferrin (Petibois and Déléris, 2003).

Long-term effects include increased resting triglyceride concentrations (Gill et al., 2003). During exercise, there’s heightened utilization of glycolysis, glycerol, and Apo-C3, accompanied by decreased neoglucogenesis and triglyceride utilization. Changes in haptoglobin and transferrin concentrations are observed, likely due to heightened erythrocyte dysfunction (Petibois and Déléris, 2003).

These results suggest the impairment of fatty acid availability, consequent to the reduction of adipose and muscle triglyceride release (Madsen et al., 1993; Petibois and Déléris, 2003).

#### 3.4.3 Lactate kinetics and lactate threshold

A master cyclist, subjected to detraining consequent to a clavicular fracture, presented a 16.7% drop in lactate threshold (Nichols et al., 2000). After 12 weeks of inactivity, 14 swimmers showed a decline in speed at the lactate threshold (Głyk et al., 2022).

After 5 weeks of training cessation, 10 highly trained rowers exhibited a fourfold increase in blood lactate concentration compared to pre-detraining values. This change was observed during an incremental and exhaustive test on a rowing-ergometer, highlighting the impact of detraining on lactate levels in this cohort (Petibois and Déléris, 2003). Additionally, among a group consisting of 4 runners and 3 cyclists, a transition from 1.9 to 7.6 mmol/L in post-exercise blood lactate concentration was noted after 84 days of inactivity. This elevation occurred during a submaximal test with the same intensity, conducted on specific exercise equipment (treadmill for runners, cycle ergometer for cyclists) (Coyle et al., 1985). Such increases significantly contribute to compromised endurance performance.

#### 3.4.4 Muscle glycogen

In runners, cyclists and triathletes, 4 weeks of training reduction resulted in a decrease of muscle glycogen by 18% (Madsen et al., 1993). This seems to be caused by a lowering in glycogen synthesis and in the enzyme glycogen synthase’s activity (Kjaer et al., 1992; Mujika and Padilla, 2000b).

#### 3.4.5 Weight, body composition and cholesterol

Body weight and body composition are not affected by short-term detraining (Coyle et al., 1984; Houmard et al., 1993; McCoy et al., 1994; Arciero et al., 1998). However, in the period longer than 4 weeks, body weight increases considerably (DB and HS, 1972; Głyk et al., 2022).

Analyzing cholesterol change induced by training cessation, a 0.9% decrease in high-density lipoprotein (HDL) type cholesterol after 6.5 days (Godfrey et al., 2005) was identified. During the same periods of detraining, Low Density Lipoprotein (LDL) cholesterol levels were increased up to 1.6% (Hardman et al., 1998) and 2.1% (Mikines et al., 1989). Very Low-Density Lipoprotein (VLDL) cholesterol manifested opposite results: no change (Hardman et al., 1998), compared with a 50% increase after 1 week of inactivity (Gill et al., 2003).

### 3.5 Muscular detraining

#### 3.5.1 Muscle fiber characteristics

A few days after training cessation, the first signs of muscle atrophy occur with fiber denervation, with neuromuscular junction damage and with a reduction of muscle protein synthesis (Paoli and Musumeci, 2020). In this context, studies are scarce and inconsistent, yet some indicate the onset of muscle atrophy within a few days of inactivity (Houmard et al., 1992). As to the amount of oxidative fiber, results are limited and conflicting as well. In contrast to studies reporting no change in fiber composition (Houmard et al., 1992; McCoy et al., 1994), Coyle et al. showed an appreciable change in the percentage of type I and type II fibers: after 56 days from the initiation of detraining, a transition from type IIa to type IIx fibers occurred in runners and cyclists, with the latter increasing from 5% of the trained state to 19% (Coyle et al., 1985).

#### 3.5.2 Muscle capillarization

Regarding changes in capillary density resulting from inactivity, there is one study showing unchanged capillarization after 12–21-56–84 days (Coyle et al., 1984). Muscle capillary density maintenance appears to be a long-term adaptation resulting from prolonged training. Conversely, sedentary individuals who underwent 8 weeks of training exhibited a reduction in this parameter (Coyle et al., 1984). Other studies also indicate a regression after 2–3 weeks (Mujika and Padilla, 2001b), such as Houston et al. (1979) study reporting a 6% decrease after 15 days of inactivity.

#### 3.5.3 Arterial-venous oxygen difference

The decrease in VO_2_max is associated with the reduction in arterial-venous oxygen differences: this parameter appears to decrease when training cessation is prolonged for more than 3–8 weeks (Coyle et al., 1984). The reduction observed could be attributed to decreased mitochondria at the muscle level. However, other contributing factors such as reduced muscle blood flow or altered blood transit time in the capillaries cannot be excluded (Mujika and Padilla, 2001a). According to Coyle et al., initial decline in VO_2_max is partially caused by the decrease in stroke volume, while the decrease in arterial-venous oxygen difference is responsible for the loss of VO_2_max observed between the thirds and 12th week of detraining (Coyle et al., 1984).

#### 3.5.4 Enzyme activities and GLUT4

As training cessation is prolonged, a decline in citrate synthase (CS) enzyme’s activity is identified in endurance trained subjects (Coyle et al., 1984; Houmard et al., 1992; Madsen et al., 1993; McCoy et al., 1994). This enzyme, involved in the process of cellular respiration, is responsible for both catalyzing the first reaction of the Krebs cycle, and for regulating the flow rate of the entire cycle. A 5% decrease in CS was observed following 6 days of inactivity (Coyle et al., 1984), a 28.6% CS decrease was seen after 10 days of inactivity (McCoy et al., 1994) while in the long-term detraining, after 56 days, the CS reduction was equal to 40% (Coyle et al., 1984). At the same time, reductions in the content of GLUT4 are reported. GLUT 4 is a glucose transporter primarily activated by insulin or muscle contraction. As a membrane protein present in adipocytes, myocytes, and cardiomyocytes, its main function involves facilitating the transfer of glucose from the interstitial fluid into the interior of the cell (Houmard et al., 1993). In highly trained athletes, GLUT4 protein content was reduced by 17% after 6 days of inactivity (Vukovich et al., 1996) and by 33% in the first 10 days (McCoy et al., 1994). In contrast to these results, Houmard et al. (1993) found that despite 14 days of detraining, the carrier content remained unchanged. The reduction in muscle oxidative capacity is further evident through the decrease in the activity of enzymes such as ß-hydroxyacyl-CoA dehydrogenase (Madsen et al., 1993) and succinate dehydrogenase (Houston et al., 1979; Coyle et al., 1984). The activity of the succinate dehydrogenase enzyme is estimated to have declined with a half-life of 12 days (Coyle et al., 1984).

Following 5 days of training cessation, a reduction in the activity of the enzyme glycogen synthase is seen, subsequently impacting the conversion of glucose into glycogen (Mikines et al., 1989; Madsen et al., 1993).

However, concerning the enzyme lactate ases have been observed (Houston et al., 1979; Coyle et al., 1985). In the context of glycolytic enzymes, specifically the activity of phosphorylase and phosphofructokinase enzymes, no changes have been noted.

A detraining period of 6.5 days has been shown to result in an increase in postprandial lipemia (Hardman et al., 1998).

#### 3.5.5 Other detraining characteristics

The decrease in oxidative enzymes results in diminished ATP production at the mitochondrial level, occurring shortly after the cessation of training (Mujika and Padilla, 2001a). Specifically, the activity of citrate synthase decreased from 10.0 to 7.7 mol kg during the initial 3 weeks without training. Subsequently, this decline continued, reaching 6.0 mol kg over the following 8 weeks, and eventually stabilized (Coyle et al., 1985).

After 4 weeks of reduced training, there may be an impaired transport of magnesium from the extracellular to the intracellular compartment. This alteration could potentially inhibit calcium release from the sarcoplasmic reticulum, thereby compromising performance (Madsen et al., 1993).

Myoglobin concentration appears not to be affected by detraining status (Coyle et al., 1984).

### 3.6 Hormonal detraining

Short-term hormonal detraining is marked by a reduction in insulin sensitivity (Mikines et al., 1989; Houmard et al., 1993; Vukovich et al., 1996) and an increase in the area under the curve of insulin response (Houmard et al., 1993; McCoy et al., 1994; Arciero et al., 1998).

Glucagon, cortisol and growth hormone (GH) levels appear to remain unchanged following a 5-day detraining period (Hardman et al., 1998) as well as after 4–6 weeks (Kjaer et al., 1992).

Despite 5 weeks of inactivity, the adrenal medulla maintains the ability to secrete epinephrine, although the reversibility of this phenomenon remains uncertain (Kjaer et al., 1992). Conversely, another study notes lowered plasma concentrations of epinephrine and norepinephrine during exercise at the same relative intensity in detrained individuals compared to trained ones (Coyle et al., 1985). The unaltered secretion capacity might partly stem from genetic predisposition and a gradual reversion of adaptations induced by training stimuli (Kjaer et al., 1992).

## 4 Discussion

This present review conducted a comprehensive analysis and synthesis of the data extracted from scientific literature, focusing on alterations in training-induced adaptations at physiological, anatomical, and performance levels among endurance athletes after a period of detraining.

Detraining, characterized as the partial or complete loss of adaptations brought about by training stimuli due to reduced or complete cessation of physical activity, leads to cardiorespiratory, muscular, metabolic, and hormonal changes in both the short-term (≤4 weeks) and long-term (>4 weeks).

Insufficient training stimulus for an endurance athlete causes, in the short-term period, a decrease in VO_2_max, blood volume and plasma volume. At the cardiac level, reductions in mass, size and thickness of the left ventricle are observed following a period of training cessation of more than 4 weeks; discordant results are found, however, regarding atrial and right ventricular remodeling.

Short-term inactivity affects the cerebrovascular system by leading to diminished blood flow in specific regions of the brain. Regarding physiological parameters, blood pressure and heart rate increase, while cardiac output decreases.

At the metabolic level, there are declines in lactate threshold and increases in post-exercise lactate concentration, body weight and RER, with a greater dependence on carbohydrates. There are reductions in muscle glycogen and changes in glucose, triglycerides, glycerol and Apo-C3.

In terms of muscle impact, the cessation of training leads to decreases in arterial-venous oxygen and GLUT4 transporter disparities, unchanged myoglobin levels, and varying outcomes regarding alterations in muscle fiber and capillary density.

Short-term hormonal detraining is marked by decreased insulin sensitivity, leading to increased HOMA-IR (a systemic insulin resistance biomarker). Notably, glucagon, growth hormone (GH), and cortisol levels remain unchanged, while findings regarding epinephrine secretion are contradictory. Due to the reversal of training-induced adaptations, endurance performance becomes compromised within just a few weeks after the commencement of the training cessation period.

While the previously mentioned results primarily focus on assessing the decline resulting from complete detraining involving the total cessation of endurance activity, only a limited number of studies have attempted to examine the impact of a partial reduction in training load (in terms of both volume and intensity) on certain physiological and performance parameters. In short-term training reduction protocols, maintaining VO_2_max levels appears achievable when combining a minimal volume of low-intensity training with at least one weekly moderate-to-high intensity training session (García-Pallarés et al., 2009). Conversely, a minor decline may manifest during short intervals when high-intensity training volume is omitted or during extended training transition phases (Almquist et al., 2020). The variability of sub-maximal endurance performance parameters is influenced by the type of training reduction protocol employed. Further research should focus on exploring various experimental training reduction protocols, encompassing different time durations, manipulation of training variables such as volume, frequency, and intensity distribution at varying percentage reductions and combinations. This approach aims to uncover and compare outcomes with the changes already documented in response to short- or long-term training cessation periods. This could prove advantageous for researchers, sport scientists, and endurance coaches, as it could shed light on optimal strategies and training guidelines for a seamless transition from a competitive season to a new general preparation training phase. Such insights could effectively mitigate the physiological and performance declines typically associated with training cessation or drastic reductions.

The present work has limitations. Firstly, the available data for the studied population are limited. Studies often encounter challenges in recruiting athletes willing to suspend their training for extended periods. Furthermore, most of the considered endurance athletes are male, and the distinct physiological characteristics between male and female genders may potentially impact alterations in various parameters and consequently, the study outcomes. Additionally, a few of the included studies are not of recent publication.

### 4.1 Return to fitness

The return to fitness following a period of training cessation is a pivotal phase for endurance athletes. Achieving a successful return requires employing effective resistance training methods, considering factors such as timing, duration, reasons for the training suspension, as well as the athletes’ level and experience. Utilizing both internal and external training load measurements for monitoring training load can assist in making informed decisions regarding rehabilitation progressions and strategies for returning to sport (Taylor et al., 2021).

Two notable endurance training methods, moderate to heavy steady-state training and severe high-intensity training, have been extensively studied for their efficacy in expediting the return to fitness. Gibala et al. underscore the significance of low-volume, high-intensity interval training (HIIT) in fostering mitochondrial biogenesis and skeletal muscle adaptation, critical for enhancing endurance athletic performance (Gibala et al., 2012). This approach, characterized by short bursts of intense exercise interspersed with brief periods of rest or lower-intensity activity, has the potential to induce significant physiological and performance adaptations, thereby expediting the return to fitness following a prolonged detraining period (Gibala and Jones, 2013; Coates et al., 2023).

An approach involving two to three weekly sessions, spanning 4–8 weeks, performed at very high intensities (around 10%–15% of the weekly total training volume) has been recommended as an effective means to enhance physiological performance for elite athletes engaged in highly intense endurance events. (Gibala and Jones, 2013). Regrettably, there is a lack of comprehensive understanding regarding the optimal training volume and intensity distribution required to retrain endurance athletes following different periods of training interruptions. Future studies ought to emphasize implementing diverse endurance training protocols aimed at enhancing fitness levels concerning both physiological parameters and performance profiles, while considering different types of detraining.

### 4.2 Age relative effects of detraining

The effects of endurance detraining differ between younger (senior) and older (master) athletes due to age-related physiological changes. Research indicates that in younger athletes, detraining leads to a swift decline in VO_2_max and a reduction in endurance capacity (Petibois and Déléris, 2003). Moreover, master athletes display diminished glycation and mechanical stress in their connective tissue, potentially playing a role in their capacity to sustain endurance levels despite experiencing detraining (Couppé et al., 2014). These findings indicate that while senior athletes undergo notable declines in cardiovascular function and endurance capacity following detraining, master athletes may sustain certain physiological adaptations acquired through lifelong training, potentially mitigating the effects of detraining.

Giada et al. were among the initial authors to investigate the impact of long-term detraining (2 months) on various cardiovascular and metabolic parameters. They compared a group of young amateur cyclists (19–25 years) with older experienced ones (50–65 years). Upon 2-month detraining, physiological variables like HRmax, VO_2_max, SBPmax, and DBPmax did not display significant differences between the two groups. However, younger athletes exhibited a smaller decline in maximal cycling power output (Wmax) during the test compared to the older athletes (−3.5% vs. −8.8%, respectively). It is important to note that the comparison in this study did not consider differences in in-season training variables between the two groups.

There is limited comprehensive knowledge regarding the effects of detraining on cardiorespiratory, metabolic, muscular, and hormonal aspects across diverse age groups. An essential consideration involves evaluating the duration and type of detraining, along with the athletes’ prior experience and fitness level before discontinuation of endurance training.

Subsequent studies should comprehensively assess the impact of age on multiple functional assessment and performance parameters after detraining of varying durations. This approach will aid in elucidating differences among different age groups and their responses to detraining.

## 5 Practical application

Even brief interruptions in physical activity can prompt numerous alterations in physiological, anatomical, and performance parameters. A considerable duration of re-training is essential for the body to restore its pre-detraining conditions. Hence, understanding the repercussions of detraining in endurance athletes can prove invaluable for coaches and athletic trainers, aiding them in identifying the most efficacious strategies to mitigate its impact.

In the future, fostering collaboration between coaches and researchers will be essential to bring evidence to the current literature. This synergy between the practical expertise of coaches and the scientific insights of researchers will aid in addressing existing gaps at various levels, including cardiac-atrial, cerebrovascular, tendon-articular, and muscle fiber aspects after training cessation periods.

Further research should focus on investigating and testing various training reduction protocol interventions to document and compare the outcomes of the physiological and performance variables.

## Data Availability

The original contributions presented in the study are included in the article/Supplementary Material. Original datasets are available in a publicity accessible repository: https://zenodo.org/uploads/10468446. Further inquiries can be directed to the corresponding author.
