# Supplementary material for: Cardiorespiratory and metabolic consequences of detraining in endurance athletes
Source: Front Physiol. 2024 Jan 22;14:1334766. doi: 10.3389/fphys.2023.1334766 (PMC10853933; doi:10.3389/fphys.2023.1334766)
Supplement: Supplementary file 1 [file Table1.docx]

**Supplementary Table 1**

Extraction of data of the included studies

*Cardiorespiratory Detraining*

| **Study** | **De- training type** | **Dura- tion (days)** | **Parameters** | **Unit of measure** | **Pre-detraining** | **Post-detraining** | **%** |
| --- | --- | --- | --- | --- | --- | --- | --- |
| **Houmard et al. (1992)** | Total cessation | 14 | VO2max | ml*kg^-1^*min^-1^ | 61.6 ± 2.0 | 58.7 ± 1.8 | -4.6 |
|  |  |  | HRmax | Beats*min^-1^ | 198 ± 3 | 207 ± 3 | +4.7 |
|  |  |  | HRsubmax | Beats*min^-1^ | 173 ± 4 | 184 ± 3 | +6.3 |
|  |  |  |  | Beats*min^-1^ | 184 ± 3 | 195 ± 4 | +6 |
|  |  |  | VE | L*min^-1^ | 126.3 ± 6.1 | 120.3 ± 6.1 | -4.7 |
|  |  |  | Hematocrit | % | 41.4 ± 1.1 | 43 ± 0.9 | +3.8 |
|  |  |  | Resting Hb | mg% | 12.1 ± 0.5 | 12.5 ± 0.7 | +3.3 |
|  |  |  | TTE | min | 13.0 ± 0.5 | 11.8 ± 0.5 | -9.2 |
|  |  |  | PV | % | NA | NA | -5.1 |
|  |  | 12 | VO2max | L*min^-1^ | 4.22 ± 0.31 | 3.93 ± 0.27 | -7 |
| **Coyle et al.** | Total | 21 |  |  |  | 3.94 ± 0.30 | -7 |
| **(1984)** | cessation | 56  84 |  |  |  | 3.67 ± 0.25  3.56 ± 0.23 | -14  -16 |
|  |  |  | Qmax | L*min^-1^ | 27.8 ± 1.5 | 26 ± 1.7 | -7 |
|  |  |  |  |  |  | 25.5 ± 1.7 | -8 |
|  |  |  |  |  |  | 25.2 ± 1.1 | -9 |
|  |  |  |  |  |  | 25.2 ± 1.2 | -10 |
|  |  |  | SV | ml | 148 ± | 134 ± 9 | -10 |
|  |  |  |  |  |  | 131 ± 9 | -11 |
|  |  |  |  |  |  | 127 ± 6 | -14 |
|  |  |  |  |  |  | 129 ± 6 | -13 |
|  |  | 5 | VO2max | ml*kg^-1^*min^-1^ | 49.8 ± 1.1 | 51 | +2.4 |
| **Doherty RA** | Total | 10 |  |  |  | 46 | -7.6 |
| **et al. (2003)** | cessation | 15 |  |  |  | 48.8 ± 1.3 | -2 |
|  |  |  | Qmax | L*min^-1^ | 18.2 | 19 | +4.4 |
|  |  |  |  |  |  | 18.3 | +0.5 |
|  |  |  |  |  |  | 18.5 | +1.6 |
|  |  |  | SVmax | ml*beat^-1^ | 93.5 | 96.7 | +3.4 |
|  |  |  |  |  |  | 92.7 | -0.9 |

|  |  |  |  |  |  | | 93.7 | | +0.2 | |
| --- | --- | --- | --- | --- | --- | --- | --- | --- | --- | --- |
|  |  |  | HRmax | Beats*min^-1^ | 195 | | 197  198  199 | | +1  +1.5  +2.1 | |
|  |  | 21 |  | | Up | Su | Up | Su | Up | Su |
| **Martin et al.** | Total | 56 |  |  | -right | -pine | -right | -pine | -right | -pine |
| **(1986)** | cessation | 84 | VO2max | L*min^-1^ | 1.52 ± 0.12 | 1.4 ± 0.16 | 1.51 ± 0.12 | 1.38 ± 0.17 | -0.7 | -1.4 |
|  |  |  |  |  |  |  | 1.49 ± 0.14 | 1.4 ± 0.18 | -2 | 0 |
|  |  |  |  |  |  |  | 1.52 ± 0.14 | 1.51 ± 0.18 | 0 | +7.8 |
|  |  |  | Qsubmax | L*min^-1^ | 16.3 ± 0.8 | 16.4 ± 1.0 | 16.1 ± 1.2 | 16.9 ± 1.2 | -1.2 | +3 |
|  |  |  |  |  |  |  | 15.1 ± 1.0 | 18.1 ± 1.9 | -7.4 | +10.4 |
|  |  |  |  |  |  |  | 16.7 ± 0.7 | 18.2 ± 1.4 | +2.5 | +11.7 |
|  |  |  | HRsubmax | Beats*min^-1^ | 111 ± 6 | 107 ± 6 | 115 ± 7 | 112 ± 6 | +3.6 | +4.6 |
|  |  |  |  |  |  |  | 124 ± 5 | 123 ± 7 | +11.7 | +13.7 |
|  |  |  |  |  |  |  | 123 ± 8 | 119 ± 7 | +9.7 | +10.1 |
|  |  |  | TPR | dyn*s*cm^-5^ | 424 ± 14 | 454 ± 21 | 464 ± 16 | 454 ± 33 | +9.4 | 0 |
|  |  |  |  |  |  |  | 496 ± 28 | 425 ± 46 | +17 | -6.4 |
|  |  |  |  |  |  |  | 466 ± 13 | 413 ± 32 | +9.9 | -2.6 |
|  |  |  | Mean BP | mmHg | 86 ± 3 | 92 ± 3 | 93 ± 5 | 94 ± 1 | +8.1 | +2.2 |
|  |  |  |  |  |  |  | 92 ± 2 | 91 ± 2 | +7 | -1.1 |
|  |  |  |  |  |  |  | 96 ± 3 | 93 ± 2 | +11.6 | +1.1 |
|  |  |  | SV | ml | 151 | 155 |  | 149 |  | -3.9 |
|  |  |  |  |  |  |  | 136 | 149 | -9.9 | -3.9 |
|  |  |  |  |  |  |  | 129 | 144 | -14.6 | -7.1 |
|  |  | 21 | LVM | g | 154 | | 124 | | -19.5 | |
|  |  | 56 |  |  |  | | 124 | | -19.5 | |
|  |  |  | LV PWT | cm | 0.8 | | 0.7 | | -12.5 | |
|  |  |  |  |  |  | | 0.6 | | -25 | |
|  |  |  | LVEDD | cm | 5.1 | 5.4 | 4.6 | 5.1 | -9.8 | -5.6 |
|  |  |  |  |  |  |  | 4.5 | 5.1 | -11.8 | -5.6 |
|  |  | 21 | LVESD | cm | 2.9 | 3.1 | 2.7 | 3.1 | -6.9 | 0 |
| **Coyle et al. (1986)** | Total cessation | 14-28 | VO2max ex | L*min^-1^ | 4.42 ± 0.17 | | 4.16 ±0.16 | | -5.9 | |
|  |  |  | SV | ml | 166 ± 8 | | 146 ± 6 | | -12 | |
|  |  |  | BV | ml | 5.18 ± 0.17 | | 4.69 ±0.13 | | -9.5 | |
|  |  |  | PV | ml | 2.9 ± 0.13 | | 2.5 ±0.11 | | -13.8 | |
|  |  |  | HR | Beats*min^-1^ | 126 ± 4 | | 140 ± 3 | | +11.1 | |
|  |  |  | TPR | dyn*s*cm^-5^ | 388 ± 30 | | 418 ± 21 | | +7.7 | |

|  |  |  | Mean BP | mmHg | 98 ± 3 | 105 ± 3 | +7.1 |
| --- | --- | --- | --- | --- | --- | --- | --- |
|  |  |  | TTE | min | 9.13 ± 0.47 | 8.44 ± 0.3 | -7.6 |
|  |  | 10 | VO2max | L*min^-1^ | 4.3 ± 0.46 | 4.23 ± 0.45 | -1.6 |
| **Cullinane et** | Total |  |  |  |  |  |  |
|  |  |  | SBP rest | mmHg | 115 ± 10 | 115 ± 11.1 | 0 |
| **al. (1986)** | cessation |  |  |  |  |  |  |
|  |  |  | DBP rest | mmHg | 75 ± 8.1 | 72 ± 9.0 | -4 |
|  |  |  | HRmax | Beats*min^-1^ | 183 ± 5.1 | 192 ± 5.2 | +4.9 |
|  |  |  | HRrest | Beats*min^-1^ | 52 ± 9 | 49 ± 7.4 | -5.8 |
|  |  |  | SV | ml | 115 ± 18.8 | 112 ± 26.3 | -2.6 |
|  |  |  | LVM | g | 227 ± 47.8 | 223 ± 37.2 | -1.8 |
|  |  |  | IVS ED-ES | mm | 9 ± 2.0 | 9 ± 1.7 | 0 |
|  |  |  |  |  | 12 ± 2.0 | 12 ± 2.1 | 0 |
|  |  |  | LV PWT ED-ES | mm | 9 ± 0.8 | 9 ± 1.2 | 0 |
|  |  |  |  |  | 17 ± 1.5 | 16 ± 2.1 | -5.9 |
| **Pelliccia et al. (2002)** | Total cessation | 365-4747 | LVEDD | mm | 61.2 ± 2.9 | 57.2 ± 3.1 | -6.5 |
|  |  |  | LVESD | mm | 39.4 ± 2.9 | 37.5 ± 2.7 | -1.9 |
|  |  |  | LV PWT | mm | 10.8 ± 0.8 | 9.5 ± 0.7 | -12 |
|  |  |  | Max LV wall  thickness | mm | 12 ± 1.3 | 10 ± 0.8 | -16.7 |
|  |  |  | LVM normalized to  height | g | 194 ± 25 | 140 ± 21 | -27.8 |
|  |  |  | LA | mm | 39.7 ± 3.2 | 39.3 ± 3.9 | -1 |
| **Chen et al. (2022)** | Total cessation | 14 | VO2max | ml*kg^-1^*min^-1^ | 64.4 ± 5.03 | 62 ± 4.84 | -3.7 |
|  |  |  | TTE | min | 15.7 ± 1.24 | 15.3 ± 1.5 | -2.5 |
|  |  |  | SVmax | ml | 139.8 ± 19.67 | 134.1 ± 18.60 | -4.1 |
|  |  |  | HRmax | Beats*min^-1^ | 194 ± 7 | 197 ± 7 | +1.5 |
|  |  | 60 | HR | Beats*min^-1^ | 167 ± 4 | 169 ± 9 | +1,1 |
| **Giada et al.** | Total |  |  |  |  |  |  |
|  |  |  | SBP | mmHg | 234 ± 17 | 230± 18 | -1,7 |
| **(1998)** | cessation |  |  |  |  |  |  |
|  |  |  | DBP | mmHg | 97 ± 11 | 94 ± 9 | -3.1 |
| **Athletes:** |  |  |  |  |  |  |  |
|  |  |  | PWTI | mm*m^-2^ | 6.3 ± 0.6 | 6.1 ± 0.6 | -3.2 |
| **older (range** |  |  |  |  |  |  |  |
|  |  |  | LAI | mm*m^-2^ | 24 ± 2 | 23 ± 2 | -4.2 |
| **50-65 years)** |  |  |  |  |  |  |  |
|  |  |  | IVSTI | mm*m^-2^ | 6.3 ± 0.6 | 6.2 ± 0.7 | -3.2 |

| **Athletes: young (range 19-25 years)** |  |  | HR | Beats*min^-1^ | 190± 9 | 189 ± 9 | -0.5 |
| --- | --- | --- | --- | --- | --- | --- | --- |
|  |  |  | SBP | mmHg | 216 ± 13 | 220 ± 14 | +1.9 |
|  |  |  | DBP | mmHg | 81 ± 10 | 77 ± 7 | -5 |
|  |  |  | PWTI | mm*m^-2^ | 6.1 ± 0.5 | 5.7 ± 0.4 | -6.6 |
|  |  |  | LAI | mm*m^-2^ | 21 ± 3 | 21 ± 2 | 0 |
|  |  |  | IVSTI | mm*m^-2^ | 6.2 ± 0.7 | 5.8 ± 0.4 | -0.2 |
| **Maron et al. (1993)** | Total cessation | 42-238 | Max LVT | mm | 13.8 ± 0.9 | 10.5 ± 0.5 | -23.9 |
|  |  |  | LVEDD | mm | 57.8 ± 2.0 | 57.1 ± 2.3 | -1.2 |
|  |  |  | LVM | g | 315 ± 19.3 | 240 ± 30.2 | -23.8 |
|  |  |  | PWT | mm | 11.3 ± 0.7 | 10.3 ± 0.9 | -8.8 |
|  |  | 7 | HR | Beats*min^-1^ | 56.5 ± 11.6 | 59.9 ± 12.6 | +6 |
| **Petretta et al.** | Partial | 21 |  |  |  | 54.9 ± 12.1 | -3 |
| **(1991)** | reduction |  | SBP | mmHg | 124.7 ± 10.2 | 122.7 ± 12.4 | -1.6 |
|  |  |  |  |  |  | 116.1 ± 11.8 | -6.9 |
|  |  |  | DBP | mmHg | 76.7 ± 7.3 | 71.9 ± 7.7 | -6.3 |
|  |  |  |  |  |  | 75.3 ± 6.1 | -1.8 |
|  |  |  | TPR | Dyn*s*cm^-5^ | 1924.3 ± 544.5 | 1597.9 ± 497.7 | -16.9 |
|  |  |  |  |  |  | 2116.2 ± 534.5 | +10 |
|  |  |  | FE | % | 52.5 ± 9.6 | 59.6 ± 10.1 | +13.5 |
|  |  |  |  |  |  | 53.8 ± 5.5 | +2.5 |
|  |  |  | PWTs | cm | 1.9 ± 0.2 | 1.74 ± 0.17 | -8.4 |
|  |  |  |  |  |  | 1.70 ± 0.18 | -10.5 |
|  |  |  | PWTd | cm | 1.08 ± 0.08 | 0.96 ± 0.11 | -11.1 |
|  |  |  |  |  |  | 1.01 ± 013 | -6.5 |
|  |  |  | LVIDs | cm | 3.35 ± 0.41 | 3.26 ± 0.37 | -2.7 |
|  |  |  |  |  |  | 3.24 ± 0.38 | -3.3 |
|  |  |  | LVIDd | cm | 5.71 ± 0.26 | 5.67 ± 0.30 | -0.7 |
|  |  |  |  |  |  | 5.45 ± 0.31 | -4.6 |
|  |  |  | IVSTs | cm | 1.74 ± 0.22 | 1.65 ± 0.17 | -5.2 |
|  |  |  |  |  |  | 1.62 ± 0.18 | -6.9 |
|  |  |  | IVSTd | cm | 1.1 ± 0.11 | 0.93 ± 0.1 | -15.5 |
|  |  |  |  |  |  | 1.03 ± 0.11 | -1.8 |
|  |  |  | LVM | g*m^2^ | 136.27 ± 19.35 | 110.89 ± 17.75 | -18.6 |
|  |  |  |  |  |  | 114.17 ± 20.44 | -16.2 |
|  |  |  | LVVs | ml*m^2^ | 32.99 ± 9 | 26.31 ± 6.9 | -20.2 |
|  |  |  |  |  |  | 28.08 ± 4.99 | -14.9 |

|  |  |  | LVVd | ml*m^2^ | 69.21 ± 12.04 | 65.68 ± 11.65  60.82 ± 7.14 | -5.1  -12.1 |
| --- | --- | --- | --- | --- | --- | --- | --- |
| **Nichols et al. (2000)** | Total cessation | 28 | VO2max | ml*kg^-1^*min^-1^ | 56.4 | 42 | -25.5 |
|  |  |  | HRmax | Beats*min^-1^ | 180 | 185 | +2.8 |
|  |  |  | [La^-^] peak | mM | 13.1 | 10.6 | -19.1 |
| **Godfrey et al. (2005)** | Total cessation | 56 | VO2peak | L*min^-1^ | 6.76 | 6.19 | -8.4 |
|  |  |  | VO2 at LT | L*min^-1^ | 5.34 | 5.26 | -1.5 |
| **Houston et al. (1979)** | Total cessation | 15 | HRmax | Beats*min^-1^ | NA | NA | +1/+5 |
|  |  |  | [La^-^] peak | mmol*L^-1^ | NA | NA | -23 |
|  |  |  | Performance Time | min | NA | NA | -25 |
| **Petibois et Déléris (2003)** | Total cessation | 7  168  329  364  532  693 | VO2max | ml*kg^-1^*min^-1^ | NA | 55 ± 6  59 ± 4  63 ± 5  54 ± 5  52 ± 6  50 ± 5 | NA |
| **Alfini et al. (2016)** | Total cessation | 10 | rCBF cerebellum R | ml*100g^-1^*min^-1^ | 43.8 ± 11.2 | 28.7 ± 6.7 | -34.5 |
|  |  |  | rCBF lingual gyrus R |  | 58.4 ± 12.4 | 35.2 ± 14.7 | -39.7 |
|  |  |  | rCBF inf. temporal gyrus L |  | 31.8 ± 15.2 | 13.6 ± 9.6 | -57.2 |
|  |  |  | rCBF inf. Parietal Lobule L |  | 123.3 ± 21.5 | 84.3 ± 20.7 | -31.6 |
|  |  |  | rCBF fusiform gyrus L |  | 50.1 ± 17.3 | 30.7 ± 12.6 | -38.7 |
|  |  |  | rCBF cerebellum L |  | 22.8 ± 14.6 | 13.7 ± 10.5 | -39.9 |
|  |  |  | rCBF Cerebellar tonsil R |  | 18.9 ± 5.9 | 10.3 ± 5.3 | -45.5 |
|  |  |  | rCBF Precuneus R |  | 55 ± 25.8 | 28.7 ± 17.5 | -47.8 |
| **Drinkwater et Horvath (1972)** | Total cessation | 90 | VO2max | ml*kg^-1^*min^-1^ | 47.8 ± 1.8 | 40.4 ± 1.0 | -15.5 |
|  |  |  | DBP | mmHg | 72.1 ± 4.2 | 69.4 ± 2.4 | -3.7 |
|  |  |  | SBP | mmHg | 113.1 ± 1.3 | 112.3 ± 2.8 | -0.7 |
|  |  |  | HRmax | Beats*min^-1^ | 191.3 ± 2.8 | 194.1 ± 4.2 | +1.5 |
|  |  |  | VEmax | L*min^-1^ | 77.5 ± 3.9 | 69.5 ± 4.1 | -10.3 |
| **Coyle et al. (1985)** | Total cessation | 12  21  56  84 | VO2submax | L*min^-1^ | 3.11 ± 0.23 | 3.17 ± 0.25  3.15 ± 0.24  3.19 ± 0.22  3.2 ± 0.25 | +1.9  +1.3  +2.6  +2.9 |

|  |  |  | HRsubmax | Beats*min^-1^ | 158 ± 3 | | 170 ± 3  174 ± 3  185 ± 5  184 ± 5 | | +7.6  +10.1  +17.1  +16.5 | |
| --- | --- | --- | --- | --- | --- | --- | --- | --- | --- | --- |
|  |  |  | VE | L*min^-1^ | 70.5 ± 5.0 | | 75.6 ± 5.7  77.6 ± 5.0  87.7 ± 6.4  90 ± 6.6 | | +7.2  +10.1  +24.4  +27.7 | |
| **Arciero et al. (1998)** | Total cessation | 7-10 | Calf BF | ml*100ml^-1^*min^-1^ | 3.17 ± 0.82 | | 3.87 ± 0.38 | | +22.1 | |
| **Houmard et al. (1993)** | Total cessation | 14 | TTE | min | 13 ± 0.5 | | 11.8 ± 0.5 | | -9.2 | |
| **Glyk et al. (2022)** | Total cessation | 84 | LT Dmax Woman Man | m*s^-1^ | 1.4 ± 0.07  1.7 ± 0.05 | | 1.4 ± 0.07  1.6 ± 0.04 | | 0  -5.9 | |
| **McConell et al. (1993)** | Partial reduction | 28 | VO2max | ml*kg^-1^*min^-1^ | 63.5 ± 1.1 | | 62.9 ± 1.3 | | -1 | |
|  |  |  | HRmax | Beats*min^-1^ | 187 ± 3 | | 191 ± 3 | | +2.1 | |
|  |  |  | HR rest | Beats*min^-1^ | 48.9 ± 2.4 | | 51.3 ± 3.3 | | +4.9 | |
|  |  |  | VE max | L*min^-1^ | 124.4 ± 4.5 | | 123.6 ± 5.7 | | -0.7 | |
|  |  |  | TTE | min | 6.05 ± 0.19 | | 6.15 ± 0.22 | | +1 | |
| **Madsen et al. (1993)** | Partial reduction | 28 | VO2max | L*min^-1^ | 4.57 ± 0.1 | | 4.54 ± 0.08 | | -0.7 | |
|  |  |  | TTE at 75%  VO2max | min | 79 ± 4 | | 62 ± 4 | | -21.5 | |
| **Garcia- Pallarés et al. (2009)** | Partial reduction | 35 |  | | Training  cessation | Training  reduction | Training  cessation | Training  reduction | Training  cessation | Training  reduction |
|  |  |  | HRmax | Beats*min^-1^ | 193 ± 6 | 189 ± 7 | 195 ± 6 | 192 ± 5 | +1 | +2.1 |
|  |  |  | [La^-^] peak | mmol*L^-1^ | 14 ± 3.3 | 13.1 ± 3.1 | 15.6 ± 4.6 | 14 ± 3.4 | +11.4 | +6.9 |
|  |  |  | VO2max | ml*kg^-1^*min^-1^ | 69.1 ± 3.9 | 68.5 ± 3.0 | 61.3 ± 2.7 | 64.6 ± 3.1 | -11.3 | -5.6 |

| **Rønnestad et al. (2014)** | Partial reduction | 56 |  | | LIT HIT | LIT | LIT HIT | LIT | LIT HIT | LIT |
| --- | --- | --- | --- | --- | --- | --- | --- | --- | --- | --- |
|  |  |  | VO2max | ml*kg^-1^*min^-1^ | 69 ± 6 | 68 ± 5 | 69 ± 8 | 65 ± 3 | 0 | -4.5 |
|  |  |  | %VO2max@4 mmol·L^-1^ [La^-^] | % | 77 ± 5 | 79 ± 4 | 78 ± 4 | 77 ± 7 | +1.3 | -2.6 |
|  |  |  | TTE 40-min | W*kg^-1^ | 3.75 | 3.8 | 3.95 | 3.55 | +5.4 | -6.4 |
|  |  |  | Relative max power (cycling) | W/kg | 5.76 ± 0.55 | 5.73 ± 0.67 | 5.77 ± 0.72 | 5.62 ± 0.50 | +0.2 | -1.8 |
| **Almquist et al. (2020)** | Partial reduction | 21 |  | | LIT SPR | LIT | LIT SPR | LIT | LIT SPR | LIT |
|  |  |  | VO2max | ml*kg^-1^*min^-1^ | 73.4 ± 4.9 | 71.3± 4.5 | 71.4 ± 4.0 | 71.0 ± 4.8 | -2.5 | -0.5 |
|  |  |  | [La^-^] 20-min all-out | mmol*L^-1^ | 4.7 ± 3.3 | 7.0 ± 2.2 | 5.6 ± 3.0 | 6.2 ± 1.6 | +19.1 | -11.5 |
|  |  |  | GE fresh (cycling) | % | 19.9 ± 1.0 | 19.1 ± 1.0 | 19.5 ± 1.0 | 19.2 ± 1.0 | −0.4 | +0.1 |
|  |  |  | GE semi-fatigued (cycling) | % | 18.9 ± 1.0 | 19.1 ± 1.0 | 18.9 ± 1.0 | 19.3 ± 1.0 | 0 | +0.2 |
|  |  |  | Relative max power (cycling) | W/kg | 6.0 ± 0.3 | 6.0 ± 0.5 | 6.0 ± 0.3 | 6.0 ± 0.4 | 0 | 0 |

BP, Blood Pressure; BV, Blood Volume; d, Diastolic; DBP, Diastolic Blood Pressure; ED, End-Diastolic; ES, End-Systolic; ex, exercise; FE, ejection fraction; GE Gross Efficiency; IVS, Interventricular Septum; IVST, Interventricular Septal Wall Thickness + I, Index; L, Left; LA, Left Atrium + I, Index; LITHIT, Low Intensity Training + High intensity training; LITSPR, Low intensity training + Sprint training; LT Dmax, Lactate-Threshold Speed; LV, Left Ventricular; LVEDD, Left Ventricular End-Diastolic Dimension; LVESD, Left Ventricular End-Systolic Dimension; LVIDd, Left Ventricular Telediastolic Diameter; LVIDs, Left Ventricular Telesystolic Diameter; LVM, Left Ventricular Mass; LVV, Left Ventricular Volume; NA, Not Available; PV, Plasma Volume; PWT, Posterior Wall Thickness + I, Index; Q, Cardiac Output; R, Right; rCBF, resting Cerebral Blood Flow; SBP, Systolic Blood Pressure; SV, Stroke Volume; THV, TPR, Total Peripheral Resistances; TTE, Time To Exhaustion;

*Metabolic Detraining*

| **Study** | **De-**  **training type** | **Duratio n (days)** | **Parameters** | **Unit of measure** | **Pre-detraining** | **Post-detraining** | **%** |
| --- | --- | --- | --- | --- | --- | --- | --- |
| **Houmard et al. (1992)** | Total cessation | 14 | RER |  | 1.03 ± 0.01 | 1.06 ± 0.01 | +2.9 ± 0.1 |
|  |  | 12 | Body Mass | kg | 68 ± 3.9 | 68.4 ± 4.1 | +0.6 |
| **Coyle et al.** | Total | 21 |  |  |  | 68.2 ± 4.3 | +0.3 |
| **(1984)** | cessation | 56 |  |  |  | 69.1 ± 4.3 | +1.6 |
|  |  | 84 |  |  |  | 70.1 ± 4.4 | +3.1 |
| **Cullinane et al. (1986)** | Total cessation | 10 | Body Mass | kg | 70.8 ± 7.4 | 69.9 ± 7.2 | -1.3 |
| **Chen et al. (2022)** | Total cessation | 14 | Body Mass | kg | 68.3 ± 7.6 | 69 ± 8.08 | +1 |
|  |  |  | Body Fat | % | 11 ± 3.12 | 11.4 ± 2.37 | +3.6 |
|  |  | 60 | Body Fat | % | 15.3 ± 2.9 | 16.8 ± 3.7 | +9.8 |
| **Giada et al. (1998)** | Total cessation |  |  |  |  |  |  |
| **Athletes: older (range 50-65 years)** |  |  |  |  |  |  |  |
|  |  |  | Body Fat | % | 14.6 ± 3.0 | 18.8 ± 3.7 | +28.8 |
| **Athletes: young (range 19-25 years)** |  |  |  |  |  |  |  |
| **Gill et al. (2003)** | Total cessation | 7 | VLDL apoB | mg*dl^-1^ | 3.04 ± 0.52 | 3.79 ± 0.32 | +24.6 |
|  |  |  | VLDL TG | mg*dl^-1^ | NA | NA | +50 |
|  |  |  | LDL | mg*dl^-1^ | 87 ± 9 | 85 ± 7 | -2.3 |
|  |  |  | HDL | mg*dl^-1^ | 48 ± 2 | 46 ± 4 | -4.2 |
|  | Total | 28 | Body Fat | % | 18.8 | 20.9 | +11.2 |

| **Nichols et al. (2000)** | cessation |  | Fat-free Mass | kg | 44 | 42.2 | -4.1 |
| --- | --- | --- | --- | --- | --- | --- | --- |
|  |  | 7 | Weight | kg | 79.1 ± 7 | 86 ± 4 | +8.7 |
| **Petibois et** | Total | 168 |  |  |  | 83 ± 4 | +4.9 |
| **Déléris (2003)** | cessation | 329 |  |  |  | 83 ± 5 | +4.9 |
|  |  | 364 |  |  |  | 86 ± 3 | +8.7 |
|  |  | 532 |  |  |  | 87 ± 5 | +10 |
|  |  | 693 |  |  |  | 88 ± 4 | +11.3 |
|  |  |  | Fat mass | kg | 10.5 ± 1.6 | 13.4 ± 2.6 | +27.6 |
|  |  |  |  |  |  | 11.3 ± 2.1 | +7.6 |
|  |  |  |  |  |  | 10.5 ± 1.6 | 0 |
|  |  |  |  |  |  | 13.8 ± 1.4 | +31.4 |
|  |  |  |  |  |  | 15.4 ± 2.0 | +46.7 |
|  |  |  |  |  |  | 17.8 ± 1.3 | +69.5 |
|  |  |  | Lactate rest | mmol*L^-1^ | NA | 1.34 ± 0.27 | NA |
|  |  |  |  |  |  | 1.4 ± 0.38 |  |
|  |  |  |  |  |  | 1.47 ± 0.31 |  |
|  |  |  |  |  |  | 1.41 ± 0.29 |  |
|  |  |  |  |  |  | 1.39 ± 0.31 |  |
|  |  |  |  |  |  | 1.31 ± 0.38 |  |
|  |  |  | TG rest | mmol*L^-1^ | NA | 1.28 ± 0.21 | NA |
|  |  |  |  |  |  | 1.13 ± 0.19 |  |
|  |  |  |  |  |  | 1.04 ± 0.17 |  |
|  |  |  |  |  |  | 1.21 ± 0.20 |  |
|  |  |  |  |  |  | 1.34 ± 0.31 |  |
|  |  |  |  |  |  | 1.47 ± 0.32 |  |
|  |  |  | Glycerol rest | mmol*L^-1^ | NA | 86 ± 11 | NA |
|  |  |  |  |  |  | 104 ± 13 |  |
|  |  |  |  |  |  | 106 ± 14 |  |
|  |  |  |  |  |  | 88 ± 9 |  |
|  |  |  |  |  |  | 78 ± 8 |  |
|  |  |  |  |  |  | 74 ± 8 |  |
|  |  |  | Fatty acids rest | mmol*L^-1^ | NA | 11.1 ± 2.1 | NA |
|  |  |  |  |  |  | 10.8 ± 2.0 |  |
|  |  |  |  |  |  | 10.9 ± 2.2 |  |
|  |  |  |  |  |  | 11 ± 2.0 |  |
|  |  |  |  |  |  | 10.8 ± 2.2 |  |
|  |  |  |  |  |  | 11.5 ± 2.3 |  |

|  |  |  | Apo-C3 rest | g*L^-1^ | NA | 0.11 ± 0.02 | NA |
| --- | --- | --- | --- | --- | --- | --- | --- |
|  |  |  |  |  |  | 0.12 ± 0.02 |  |
|  |  |  |  |  |  | 0.13 ± 0.02 |  |
|  |  |  |  |  |  | 0.12 ± 0.02 |  |
|  |  |  |  |  |  | 0.11 ± 0.03 |  |
|  |  |  |  |  |  | 0.11 ± 0.03 |  |
|  |  |  | Transferrin rest | g*L^-1^ | NA | 3 ± 0.70 | NA |
|  |  |  |  |  |  | 3.01 ± 0.66 |  |
|  |  |  |  |  |  | 2.88 ± 0.56 |  |
|  |  |  |  |  |  | 2.91 ± 0.71 |  |
|  |  |  |  |  |  | 2.99 ± 0.58 |  |
|  |  |  |  |  |  | 2.93 ± 0.57 |  |
|  |  |  | Haptoglobin rest | g*L^-1^ | NA | 1.69 ± 0.50 | NA |
|  |  |  |  |  |  | 1.82 ± 0.61 |  |
|  |  |  |  |  |  | 1.79 ± 0.57 |  |
|  |  |  |  |  |  | 1.74 ± 0.48 |  |
|  |  |  |  |  |  | 1.67 ± 0.51 |  |
|  |  |  |  |  |  | 1.7 ± 0.49 |  |
|  |  |  | Amino acids rest | mmol*L^-1^ | NA | 0.28 ± 0.07 | NA |
|  |  |  |  |  |  | 0.31 ± 0.09 |  |
|  |  |  |  |  |  | 0.32 ± 0.10 |  |
|  |  |  |  |  |  | 0.28 ± 0.07 |  |
|  |  |  |  |  |  | 0.31 ± 0.11 |  |
|  |  |  |  |  |  | 0.32 ± 0.10 |  |
|  |  |  | Glucose rest | mmol*L^-1^ | NA | 4.81 ± 0.39 | NA |
|  |  |  |  |  |  | 4.86 ± 0.32 |  |
|  |  |  |  |  |  | 4.78 ± 0.56 |  |
|  |  |  |  |  |  | 4.51 ± 0.41 |  |
|  |  |  |  |  |  | 4.57 ± 0.37 |  |
|  |  |  |  |  |  | 4.64 ± 0.31 |  |
|  |  |  | Albumin rest | g*L^-1^ | NA | 42.2 ± 5.1 | NA |
|  |  |  |  |  |  | 41.2 ± 4.5 |  |
|  |  |  |  |  |  | 41.1 ± 4.7 |  |
|  |  |  |  |  |  | 43 ± 4.9 |  |
|  |  |  |  |  |  | 41.5 ± 4.2 |  |
|  |  |  |  |  |  | 40.1 ± 3.9 |  |
| **Drinkwater**  **et Horvath (1972)** | Total cessation | 90 | Weight | kg | 50.81 | 52.2 | +2.7 |
|  |  | 10 | Glucose | mg*dl^-1^ | 95 | 89 ± 1 (0.2mU*min^-1^*kg^-^ | -6.3 |
|  | Total |  |  |  |  | 1) | -6.3 |

| **Mikines et al. (1989)** | cessation |  |  |  |  | 89 ± 1 (0.3mU*min^-1^*kg^-^  1)  89 ± 1 (5mU*min^-1^*kg^-1^) | -6.3 |
| --- | --- | --- | --- | --- | --- | --- | --- |
|  |  |  | Glycerol | μmol/l | 35 | 10 ± 2(0.2mU*min^-1^*kg^-^  1)  5 ± 1(0.3mU*min^-1^*kg^-1^)  6 ± 2(5mU*min^-1^*kg^-1^) | -71.4  -85.7  -82.8 |
|  |  |  | Lactate | mmol*l^-1^ | 0.58 | 0.5 ± 00.6 (0.2mU*min^-1^*kg^-^  1)  0.63 ± 0.06 (0.3mU*min^-^  ^1^*kg^-1^)  1.68 ± 0.38 (5mU*min^-1^*kg^-^  1) | -13.8  +8.6  +189.7 |
| **McCoy et al. (1994)** | Total cessation | 10 | Glucose | mmol*l^-1^ | 4.8 ± 0.2 | 4.9 ± 0.2 | +2.1 |
|  |  |  | Weight | kg | 75.5 ± 0.9 | 76.3 ± 0.5 | +1.1 |
|  |  |  | BMI | kg*m^2^ | 22.5 ± 0.3 | 22.9 ± 0.4 | +1.8 |
|  |  |  | Body Fat | % | 10.9 ± 0.1 | 10.3 ± 0.8 | -5.5 |
|  |  | 12 | Lactate rest | mmol | 0.8 ± 0.1 | 0.9 ± 0.1 | +12.5 |
| **Coyle et al.** | Total | 21 |  |  |  | 0.8 ± 0.1 | 0 |
| **(1985)** | cessation | 56 |  |  |  | 0.9 ± 0.1 | +12.5 |
|  |  | 84 |  |  |  | 0.8 ± 0.1 | 0 |
|  |  |  | Lactate ex | mmol | 1.9 ± 0.2 | 3.8 ± 0.4 | +100 |
|  |  |  |  |  |  | 4.3 ± 0.3 | +126 |
|  |  |  |  |  |  | 7.2 ± 1.1 | +278 |
|  |  |  |  |  |  | 7.6 ± 0.6 | +300 |
|  |  |  | RER |  | 0.93 ± 0.01 | 0.97 ± 0.01 | +4.3 |
|  |  |  |  |  |  | 0.98 ± 0.01 | +5.4 |
|  |  |  |  |  |  | 0.99 ± 0.01 | +6.5 |
|  |  |  |  |  |  | 1 ± 0.01 | +7.5 |
|  |  |  | Glycerol rest | mmol | 0.05 ± 0.01 | 0.04 ± 0.01 | -20 |
|  |  |  |  |  |  | 0.05 ± 0.01 | 0 |
|  |  |  |  |  |  | 0.04 ± 0.01 | -20 |
|  |  |  |  |  |  | 0.04 ± 0.01 | -20 |
|  |  |  | Glycerol ex | mmol | 0.07 ± 0.01 | 0.06 ± 0.01 | -14.3 |
|  |  |  |  |  |  | 0.07 ± 0.01 | 0 |
|  |  |  |  |  |  | 0.07 ± 0.01 | 0 |

|  |  |  |  |  |  | 0.07 ± 0.01 | 0 |
| --- | --- | --- | --- | --- | --- | --- | --- |
|  |  |  | FFA rest | mmol | 0.45 ± 0.08 | 0.41 ± 0.11  0.46 ± 0.07  0.44 ± 0.09  0.36 ± 0.11 | -8.9  +2.2  -2.2  -20 |
|  |  |  | FFA ex | mmol | 0.29 ± 0.08 | 0.28 ± 0.07  0.29 ± 0.07  0.24 ±0.08  0.27 ± 0.09 | -3.4  0  -17.2  -6.9 |
| **Hardman et al. (1998)** | Total cessation | 6.5 | LDL | mM | 3.01 ± 0.2 (15h after ex) | 3.12 ± 0.19 | +3.7 |
|  |  |  | HDL | mM | 1.17 ± 0.06(15h after ex) | 1.13 ± 0.09 | -3.4 |
|  |  |  | VLDL | mM | 0.39 ± 0.07(15h after ex) | 0.5 ± 0.05 | +28.2 |
| **Arciero et al. (1998)** | Total cessation | 7-10 | Weight | kg | 78.9 ± 1.1 | 78.7 ± 1 | -0.3 |
| **Houmard et al. (1993)** | Total cessation | 14 | AUC-Glucose | mg*dl^-1^*h^-1^ | 236.4 ± 8.4 | 271.4 ± 10.4 | +14.8 |
| **Vukovich et al. (1996)** | Total cessation | 6 | Plasma Glucose | mg*dl^-1^ | 84 ± 3 | 86 ± 2 | +2.4 |
|  |  |  | Weight | kg | 64.6 ± 2.9 | 66.3 ± 2.7 | +2.6 |
| **Mikines et al. (1989)** | Total cessation | 5 | Lactate rest | mmol*L^-1^ | 0.72 ± 0.12 | 1 ± 0.12 | +38.9 |
|  |  |  | FFA | mmol*L^-1^ | 0.29 ± 0.05 | 0.25 ± 0.04 | -13.8 |
|  |  |  | Glycerol | mmol*L^-1^ | 0.027 ± 0.003 | 0.023 ± 0.004 | -14.8 |
| **Glyk et al. (2022)** | Total cessation | 84 | Weight Woman  Man | kg | 64.9 ± 6.71  79.4 ± 5.93 | 66.2 ± 8.05  83 ± 8.39 | +2  +4.5 |
|  |  |  | Body Fat Woman  Man | % | 19.6 ± 5.17  9.7 ± 2.20 | 20.9 ± 6.43  11.5 ± 3.44 | +6.6  +18.6 |
| **McConell et al. (1993)** | Partial reduction | 28 | Weight | kg | 71.0 ± 2.9 | 71.8 ± 3.1 | +1.1 |
|  |  |  | Body fat | % | 10.4 ± 1.0 | 11.8 ± 1.1 | +13.4 |

|  |  |  |  |  |  | |  | |  | |
| --- | --- | --- | --- | --- | --- | --- | --- | --- | --- | --- |
| **Madsen et al. (1993)** | Partial reduction | 28 | Muscle glycogen | mmol*kg^-1^ | 532 ± 36 | | 434 ± 46 | | -18.3 | |
|  |  |  | RER |  | 0.91 ± 0.01 | | 0.89 ± 0.01 | | -2.2 | |
| **Rønnestad et al. (2014)** | Partial reduction | 56 | Weight | kg | LIT  HIT | LIT | LIT  HIT | LIT | LIT  HIT | LIT |
|  |  |  |  |  | 77.8 ± 5.5 | 74.3 ± 8.1 | 77.9 ± 6.6 | 73.2 ± 8.4 | +0.1 | -1.4 |
| **Almquist et al. (2020)** | Partial reduction | 21 | Weight | kg | LIT  SPR | LIT | LIT  SPR | LIT | LIT  SPR | LIT |
|  |  |  |  |  | 73.6 ± 9.0 | 73.1 ± 4.8 | 74.2 ± 9.4 | 73.7 ± 4.9 | +0.7 ± 1.0 | +0.8 ± 1.0 |

AUC, Area Under Curve; FFA, Free Fatty Acid; HDL, High-Density Lipoprotein; LDL, Low Density Lipoprotein; LITHIT, Low Intensity Training + High intensity training; LITSPR, Low intensity training + Sprint training; NA, Not Available; RER, Respiratory Exchange Ratio; VLDL, Very Low-Density Lipoprotein

*Muscular Detraining*

| **Study** | **De**  **-training type** | **Duratio n (days)** | **Parameters** | **Unit of measure** | **Pre-detraining** | **Post-detraining** | **%** |
| --- | --- | --- | --- | --- | --- | --- | --- |
| **Houmard et al. (1992)** | Total cessation | 14 | Citrate Synthase | μmol*(min*g)^-1^ | 41.0 ± 3.6 | 30.6 ± 2.8 | -25.4 |
|  |  |  | Fiber | % | 61 I, 35 IIa, 4 IIx | 61 I, 35 IIa, 4 IIx | 0 |
|  |  |  | CS area fiber I | μm^2^*10^2^ | 44.3 ± 2.7 | 47.3 ± 2.1 | +6.8 |
|  |  |  | CS area fiber II | μm^2^*10^2^ | 49.2 ± 3.3 | 48.3 ± 2.6 | -1.8 |
| **Coyle et al. (1984)** | Total cessation | 6  12  21  56  84 | Capillary density | cap/mm^2^ | 464 ± 12 | 497 ± 11  467 ± 11  450 ± 12  438 ± 20  476 ± 30 | +7.1  +0.6  -3  -5.6  +2.6 |
|  |  |  | Citrate Synthase | mol*kg protein^-1^  *h-1 | 10 ± 0.6 | 9.5 ± 0.7  8.3 ± 0.6  7.7 ± 0.4  6.0 ± 0.4  6.1 ± 0.5 | -6.3  -17.1  -23.7  -40.6  -39.6 |

|  |  |  | SDH | mol*kg protein^-1^  *h-1 | 4.43 ± 0.27 | 4.04 ± 0.22  3.61 ± 0.13  3.37 ± 0.23  2.73 ± 0.08  2.99 ± 0.22 | -8.8  -18.5  -23.9  -38.4  -32.5 |
| --- | --- | --- | --- | --- | --- | --- | --- |
|  |  |  | Myoglobin | (mg*g protein^-1^) | 43.3 ± 4.4 | 43.6 ± 5.3  43.6 ± 5.1  41 ± 3.4  40.1 ± 2.1  40.7 ± 4.0 | +0.69  +0.69  -5.3  -7.4  -6 |
|  |  |  | a-v̄̄̄̄ O2 diff | ml*100ml^-1^ | 15.1 ± 0.5 | 15.1 ± 0.4  15.4 ± 0.4  14.5 ± 0.5  14.1 ± 0.5 | 0  +2  -4  -7 |
| **Doherty RA et al. (2003)** | Total cessation | 5  10  15 | a-v̄̄̄̄ O2 diff | ml*100ml^-1^ | 15.7 | 15.5  14.6  15.8 | -1.3  -7  +0.6 |
| **Madsen et al. (1993)** | Partial reduction | 28 | Capillary density | Cap/fiber | 2.31 ± 0.10 | 2.28 ± 0.09 | -1.3 |
|  |  |  | Citrate Synthase | mmol*kg dry wt^-1^  *min^-1^ | 44.7 ± 1.5 | 43.7 ± 2.3 | -2.2 |
|  |  |  | Beta-hydroxyacil-  CoA dehydrogenase | mmol*kg dry wt^-1^  *min^-1^ | 35.9 ± 1.6 | 31.6 ± 1.6 | -12 |
|  |  |  | Fiber I IIa  IIx | % | 58 ± 3  23 ± 3  19 ± 3 | 57 ± 4  25 ± 4  18 ± 3 | -1.7  +8.7  -5.3 |
|  |  |  | Na+-K+ pumps | pmol*g^-1^ dry wt | 1.15 ± 0.48 | 1.16 ± 0.35 | +0.9 |
| **Houston et al. (1979)** | Total cessation | 15 | Fiber composition ST  FTa | % | 64.2  35.7 | 63  36.6 | -1.9  +2.5 |
|  |  |  | Capillary density | N° Capillaries per  mm^2^ | 558 | 523 | -6.3 |
|  |  |  | SDH | μmol*g(wet  weight)^-1^*min^-1^ | NA | NA | -24 |
|  |  |  | LDH | μmol*g(wet weight)^-1^*min^-1^ | NA | NA | -13 |
| **McCoy et al. (1994)** | Total cessation | 10 | Citrate Synthase | μmol*min^-1^*g^-1^ | 47.12 ± 4.75 | 33.63 ± 3.98 | -28.6 |

|  |  |  | GLUT4 | Arbitrary  Standard Units | 4.37 | 2.92 | -33.2 |
| --- | --- | --- | --- | --- | --- | --- | --- |
| **Coyle et al. (1985)** | Total cessation | 6  12  21  56  84 | Fiber I  IIa  IIx | % | 52 ± 5  43 ± 3  5 ± 1 | 54 ± 5  51 ± 5  51 ± 5  57 ± 4  55 ± 4  41 ± 4  42 ± 4  36 ± 3  24 ± 3  26 ± 3  5 ± 1  7 ± 1  13 ± 3  19 ± 3  19 ± 4 | +3.8  -1.9  -1.9  +9.6  +5.8  -4.7  -2.3  -16.3  -44.2  -39.5  0  +40  +160  +280  +280 |
| **Houmard et al. (1993)** | Total cessation | 14 | GLUT4 | Arbitrary  Absorbance units | 0.802 ± 0.101 | 0.736 ± 0.74 | -8.2 |
|  |  |  | Citrate Synthase | μmol*g^-1^*min^-1^ | 41 ± 3.6 | 30.6 ± 2.8 | -25.4 |
|  |  |  | Cross section area Fiber I  II | *10^2^μm^2^ | 58.3 ± 3.9  70.9 ± 4.9 | 55 ± 3.3  66.7 ± 4.3 | -5.7  -5.9 |
| **Vukovich et al. (1996)** | Total cessation | 6 | GLUT4 | optical density*50μg protein^-1^ | 0.24 ± 0.02 | 0.2 ± 0.2 | -16.7 |

a-v̄̄̄̄ O2 diff, Arterial-Venous Oxygen Difference; CS, Cross Sectional; LDH, Lactate dehydrogenase; NA, Not Available; SDH, succinate dehydrogenase

*Hormonal Detraining*

| **Study** | **De**  **-training type** | **Duration (days)** | **Parameters** | **Unit of measure** | **Pre-detraining** | **Post-detraining** | **%** |
| --- | --- | --- | --- | --- | --- | --- | --- |
|  | Total | 7 | Insulin | NA | NA | NA | +39 |

| **Gill et al. (2003)** | cessation |  |  |  |  |  |  |
| --- | --- | --- | --- | --- | --- | --- | --- |
| **Mikines et al. (1989)** | Total cessation | 10 | Insulin | μU*ml^-1^ | 9.3 ± 0.8 | 19.2 ± 1.6 (0.2mU*min^-^  ^1^*kg^-1^)  43.3 ± 3.2 (0.7mU*min^-^  ^1^*kg^-1^)  380 ± 18 (5 mU*min^-1^*kg^-^  1) | +106.5  +365.6  +3986 |
|  |  |  | Growth hormone | mU*l^-1^ | 6 ± 4 | 7 ± 2 (0.2mU*min^-1^*kg^-1^)  11 ± 5 (0.7mU*min^-1^*kg^-1^)  1 ± 0.3 (5mU*min^-1^*kg^-1^) | +16.7  +83.3  -66.7 |
|  |  |  | Peptide C | nmol*l^-1^ | 0.41 ± 0.03 | 0.33 ± 0.03  (0.2mU*min^-^  ^1^*kg^-1^)  0.26 ± 0.03 (0.3mU*min^-^  ^1^*kg^-1^)  0.22 ± 0.03 (5mU*min^-1^*kg^-1^) | -19.5  -36.6  -46.3 |
|  |  |  | Epinephrine | nmol*l^-1^ | 0.49 ± 0.11 | 0.6 ± 0.11 (0.2mU*min^-1^*kg^-^  1)  0.87 ± 0.22 (0.3mU*min^-^  ^1^*kg^-1^)  0.49 ± 0.11 (5mU*min^-1^*kg^-1^) | +22.4  +77.6  0 |
|  |  |  | Norepinephrine | nmol*l^-1^ | 1.07 ± 0.06 | 1.36 ± 0.06 (0.2mU*min^-^  ^1^*kg^-1^)  1.24 ± 0.12(0.3mU*min^-^  ^1^*kg^-1^)  1.42 ± 0.12(5mU*min^-1^*kg^-1^) | +27.1  +15.9  +32.7 |
|  |  |  | Cortisol | nmol*l^-1^ | 417 ± 90 | 354 ± 66 (0.2mU*min^-1^*kg^-^  1)  382 ± 67 (0.3mU*min^-1^*kg^-^  1)  300 ± 79 (5mU*min^-1^*kg^-1^) | -15.7  -8.4  -28.1 |
|  |  |  | Glucagon | nmol*l^-1^ | 13 ± 2 | 11 ± 3 (0.2mU*min^-1^*kg^-1^)  13 ± 3 (0.3mU*min^- 1^*kg^-1^)  4 ± 1 (5mU*min^-1^*kg^-1^) | -15.4  0  -69.2 |
| **McCoy et al. (1994)** | Total cessation | 10 | AUC-Insulin x 10^-3^ | pmol*l^-1^*min^-1^ | 22.4 ± 2.8 | 32.1 ± 5.9 | +43.3 |
| **Coyle et al. (1985)** | Total cessation | 21  84 | Epinephrine | pg*ml^-1^ | 244 ± 55 | 316 ± 128  487 ± 192 | +29.5  +99.6 |
|  |  |  | Norepinephrine | pg*ml^-1^ | 2.346 ± 324 | 2.521 ± 584 | +7.5 |

|  |  |  |  |  |  | 3.877 ± 806 | +65.3 |
| --- | --- | --- | --- | --- | --- | --- | --- |
| **Hardman et al. (1998)** | Total cessation | 6.5 | Insulin | μIU*ml^-1^ | 6.6 ± 0.7 (15h after ex) | 7.1 ± 1.1 | +7.6 |
| **Arciero et al. (1998)** | Total cessation | 7-10 | AUC-Insulin x 10^-3^ | μU*ml^-1^*180min^-^  1 | 2.182 ± 270 | 3.793 ± 739 | +82.1 |
| **Houmard et al. (1993)** | Total cessation | 14 | AUC-Insulin x 10^-3^ | μU**l^-1^*h^-1^ | 60.4 ± 7.1 | 78.7 ± 7.7 | +30.3 |
|  |  |  | ISI |  | 17.7 ± 1.7 | 13.5 ± 1.3 | -23.7 |
| **Vukovich et al. (1996)** | Total cessation | 6 | Insulin clearance Infusion 15mU*m^-^  ^2^*min^-1^  40mU*m^-2^*min^-1^ | ml*m^-2^*min^-1^ | 1007 ± 71.0  793 ± 27.8 | 812 ± 49.2  730.1 ± 49.2 | -19.4  -7.9 |
| **Mikines et al. (1989)** | Total cessation | 5 | Insulin  Clamp step (mM) 7  11  20 | μU*ml^-1^*min | 509 ± 61  1045 ± 217  3504 ± 956 | 867 ± 154  1278 ± 326  4475 ± 741 | +70.3  +22.3  +27.7 |
|  |  |  | Proinsulin  Clamp step (mM) 7  11  20 | pmol*L^-1^*min | 592 ± 168  1309 ± 224  1934 ± 335 | 954 ± 117  1660 ± 317  1998 ± 319 | +61.1  +26.8  +3.3 |
|  |  |  | Peptide C  Clamp step (mM) 7  11  20 | nmol*L^-1^*min | 33 ± 4  47 ± 7  70 ± 13 | 39 ± 6  55 ± 8  73 ± 9 | +18.2  +17  +4.3 |
|  |  |  | Glucagon | pmol*L^-1^ | 12.2 ± 0.7 | 12.8 ± 0.11 | +4.9 |
|  |  |  | Cortisol | nmol*L^-1^ | 482 ± 107 | 466 ± 90 | -3.3 |
|  |  |  | Growth hormone | mU*L^-1^ | 7.4 ± 3.5 | 5.6 ± 1.7 | -24.3 |
|  |  |  | Norepinephrine | nmol*L^-1^ | 1.33 ± 0.14 | 1.55 ± 0.34 | +16.5 |
|  |  |  | Epinephrine | nmol*L^-1^ | 0.51 ± 0.12 | 0.56 ± 0.11 | +9.8 |

AUC, Area Under Curve; HOMA-IR, Biomarker Of Systemic Insulin Resistance; ISI, Insulin Sensitivity Index; NA, Not Available
